# Protumorigenic Responses of CEACAM6 in *Helicobacter pylori*‐Infected Gastric Cancer Cells

**DOI:** 10.1111/jcmm.70869

**Published:** 2026-01-28

**Authors:** Debashish Chakraborty, Indrajit Poirah, Supriya Samal, Smaran Banerjee, Aranya Pal, Chandan Mahish, Subhasis Chattopadhyay, Girija Nandini Kanungo, Pusparaj Samantasinhar, Gautam Nath, Niranjan Rout, Shivaram Prasad Singh, Asima Bhattacharyya

**Affiliations:** ^1^ School of Biological Sciences National Institute of Science Education and Research (NISER) Bhubaneswar, an OCC of Homi Bhabha National Institute Khurda Odisha India; ^2^ Immuno Haematology and Blood Transfusion IMS and SUM Hospital Bhubaneswar Odisha India; ^3^ Forensic Medicine and Toxicology, IMS and SUM Hospital Bhubaneswar Odisha India; ^4^ Department of Gastroenterology Acharya Harihar Post Graduate Institute of Cancer Cuttack Odisha India; ^5^ Digestive Diseases Centre Beam Diagnostics Building Cuttack Odisha India; ^6^ Centre for Interdisciplinary Sciences (CIS) NISER, An OCC of Homi Bhabha National Institute Khurda Odisha India

**Keywords:** carcinoembryonic antigen‐related cell adhesion molecules, gastric cancer, *Helicobacter pylori*, macrophage, peripheral blood mononuclear cells, soluble factors

## Abstract

*Helicobacter pylori* poses a significant risk for gastric cancer (GC) development. *H. pylori* exploits carcinoembryonic antigen‐related cell adhesion molecules (CEACAMs) on GC cells (GCCs) to colonise the gastric epithelium. CEACAM1, CEACAM5 and CEACAM6 are known to interact with *H. pylori* . We explored the role of *H. pylori* in altering CEACAM levels in GCCs and the paracrine effect of infected GCCs on neighbouring uninfected GCCs and macrophages. *H. pylori* significantly upregulated CEACAM6. Elevated CEACAM6 in GCCs promoted cell proliferation, cell migration and cell invasion. The effect was further enhanced after infection with *H. pylori* . Similarly, soluble factors released by *CEACAM6*‐transfected GCCs promoted the tumorigenic potential of uninfected GCCs. Macrophages are crucial for GC development and progression. Therefore, it was intriguing to know how CEACAM6 could influence the polarisation of macrophages during *H. pylori* infection. To study this, we co‐cultured macrophages with either the empty vector or *CEACAM6*‐expressing GCCs and found that *H. pylori* infection increased the M2 polarisation of macrophages co‐incubated with *CEACAM6*‐expressing GCCs. In summary, CEACAM6 was found to promote GC aggressiveness and alter macrophage polarisation. This information could be harnessed to develop future therapeutics for targeting GC.

**Abbreviations**
CEACAM: carcinoembryonic antigen‐related cell adhesion molecules
GC: gastric cancer
GCC: gastric cancer cells

*H. pylori*

:
*Helicobacter pylori*

MOI: multiplicities of infection
PBMC: peripheral blood mononuclear cells
STAD: stomach adenocarcinoma
TCGA: the cancer genome atlas
TME: tumour microenvironment

## 1 Introduction

Carcinoembryonic antigen‐related cell adhesion molecules (CEACAMs) are members of the immunoglobulin superfamily. These are widely distributed molecules, expressed on epithelial cells, endothelial cells, neutrophils, macrophages, T lymphocytes and B lymphocytes [1]. Their roles in cell–cell adhesion, apoptosis, cell proliferation, angiogenesis and immune responses are well documented. Pathogens exploit host cell CEACAMs as receptors to initiate infection [2, 3]. *Helicobacter pylori* , the pathogen responsible for gastric cancer (GC), like many other Gram‐negative bacteria, utilises its adhesin protein HopQ to interact with CEACAM1, CEACAM5 and CEACAM6 expressed on the host gastric epithelium [4]. Interestingly, these CEACAMs are also upregulated by *H. pylori* infection [5]. Adhesion and colonisation of the gastric epithelium by *H. pylori* trigger pro‐inflammatory responses in GC cells (GCCs) and attracts immune cells to the infection site. This response causes the development of chronic‐active gastritis, which might gradually follow Correa's cascade to develop gastric adenocarcinoma or variably lead to other gastroduodenal pathologies [6, 7].

GC progression is critically dependent on the tumour microenvironment (TME). Macrophages are the most abundant immune cells in the TME and are linked with poor prognosis in many cancers, including GC [8, 9]. Acute infection with *H. pylori* attracts macrophages to the site of infection [10, 11]. These macrophages are mostly of the M1 phenotype and create inflammatory conditions [12]. The TME releases various cytokines such as IL‐4, IL‐10 and IL‐13 that reprogram the M1 macrophages towards the M2 phenotype which exhibits anti‐inflammatory and wound‐healing properties [13]. M2 macrophages, characterised by the surface expression of CD163, CD204 and CD206, play critical roles in cell signalling events, immune suppression, angiogenesis and epithelial to mesenchymal transition (EMT) of tumour cells [14].

CEACAMs have diverse functions and are implicated in various oncogenic signalling events, such as PI3K/AKT and MAPK pathways, thereby contributing to tumour progression [15]. High CEACAM1, CEACAM5 and CEACAM6 levels in GC correlate with poor prognosis [16]. Continuous cross‐talk of cancerous epithelial cells with macrophages and normal epithelial cells within the TME contributes to the tumour progression. Interestingly, when co‐cultured together, invasive breast cancer cells induce EMT in normal breast epithelial cells [17]. A solitary report shows that the absence of CEACAM1 in epithelial cells is associated with the induction of neoangiogenesis via modulation of M1 macrophages in breast cancer [18]. However, the influence of *H. pylori* infection‐induced epithelial CEACAMs in modulating macrophage‐ and epithelial cell response in GC remains largely unexplored.

This study evaluated the effect of *H. pylori* ‐26695 infection on CEACAMs. Among CEACAM1, CEACAM5 and CEACAM6, only CEACAM6 was found to be significantly increased upon infection. This is the first report regarding the effect of CEACAM6 after *H. pylori* infection in potentiating proliferation, migration, invasion and colony forming ability of GCCs. Interestingly, we also found that supernatants derived from infected *CEACAM6*‐overexpressing GCCs imparted similar effects on uninfected GCCs. Further, in vitro analysis confirmed that macrophages shifted towards the M2 phenotype when cocultured with *H. pylori‐*infected *CEACAM6*‐overexpressing GCCs. Analysis of *H. pylori* ‐infected human GC biopsy samples also revealed higher congregation of M2 macrophages compared to their paired controls. Collectively, our data identified the cancer‐promoting ability of CEACAM6 in the *H. pylori* ‐mediated GC and provided a foundation for future studies of CEACAM6 to develop therapeutic intervention strategies to fight against *H. pylori* ‐mediated GC.

## 2 Materials and Methods

### 2.1 Maintenance of Cell Lines and *H. pylori*

AGS (ATCC, Manassas, VA, USA), MKN45 (collected through material transfer agreement between NISER and UVA), and *H. pylori* 26695 (*cag* PAI positive) (ATCC) were maintained as described elsewhere [19]. Cell lines were infected with *H. pylori* at 20 multiplicities of infection (MOI) for 24 h, unless stated otherwise.

### 2.2 Gene Expression and Associated Studies

Analysis of transcript levels of *CEACAM1*, *CEACAM5* and *CEACAM6* in Stomach Adenocarcinoma (STAD) dataset from The Cancer Genome Atlas (TCGA) was performed in Gene Expression Profiling Interaction Analysis‐2 [20] (GEPIA2; https://gepia2.cancer‐pku.cn/, date of accession‐20th October, 2024). From The University of Alabama at Birmingham Cancer data analysis portal [21] (UALCAN; https://ualcan.path.uab.edu/), *H. pylori* specific information of the patients was retrieved from the STAD dataset (date of accession‐20th October, 2024). Stage specific analysis of transcript levels of *CEACAM1*, *CEACAM5* and *CEACAM6* was also performed in UALCAN.

### 2.3 Stable Cell Generation, Supernatant Collection and Assessment of Cytokines

25 × 10^4^ AGS cells were plated in a 96‐well dish and the cells were transfected with the empty vector or *CEACAM6* plasmid (a generous gift from the late Dr. Bernhard B. Singer, University of Essen, Germany) using Lipofectamine 3000 (Thermo Fisher Scientific) as per the manufacturer's protocol. After 48 h, the cells were grown in media containing G418 (Sigma‐Aldrich, Saint Louis, MO, USA). The selection pressure was maintained till stable colonies were obtained.

3 × 10^6^ empty vector or *CEACAM6* stably‐transfected cells with/without *H. pylori* infection were cultured in 2 mL of RPMI 1640 supplemented with 10% heat‐inactivated FBS for 24 h. Supernatants were collected after centrifugation at 5000*g* for 5 min. Human inflammation antibody array (ab134001, Abcam, MA, USA) was used to assess the cytokines present within the supernatants. All the procedures were followed as described in the manufacturer's protocol. Chemiluminescence was captured using Chemidoc XRS+ (Bio‐Rad, CA, USA). Densitometry analysis was done in ImageLab software (Bio‐Rad) and quantitation of the spots was performed as described in the manufacturer's protocol.

### 2.4 Human Gastric Biopsy Collection

Biopsy samples were collected from the antral region of the stomach of GC patients corresponding to the gastric adenocarcinoma (*n* = 3) and metastatic stages (*n* = 3). Prior consent was taken and patient identities were protected. The biopsy collection procedure complied with the Helsinki Declaration (2013), World Medical Association and was approved by the Institutional Ethics Committee for Human Research, National Institute of Science Education and Research. 4% paraformaldehyde‐fixed specimens were cryosectioned (Leica, Germany) at 5 μm thickness and used for further analysis.

### 2.5 Western Blotting

1 × 10^6^ AGS and MKN45 cells were plated in 35‐mm dishes. After infection with *H. pylori* , cell lysates were generated. SDS‐PAGE and western blotting were performed as described earlier [22]. Blots were incubated with CEACAM1 (C5‐1X/8), CEACAM5 (3E10‐3), CEACAM6 (1H7‐4B) (generous gifts from the late Dr. Bernhard B. Singer) and GAPDH (Abgenex, India) antibodies overnight at 4°C. Immunoblots were then probed with HRP‐conjugated secondary antibodies (Cell Signalling Technology, MA, USA) at room temperature. Chemiluminescence obtained after incubation with a chemiluminescent substrate kit (Thermo Fisher Scientific) was captured using Chemidoc XRS+ (Bio‐Rad). Densitometry analysis of the immunoblots was performed with ImageLab software (Bio‐Rad).

### 2.6 Human Peripheral Blood Mononuclear Cells (PBMCs)‐Derived Monocyte Culture and Differentiation

Blood from healthy donors was obtained from blood bank. Blood diluted with PBS in a ratio of 1:1 was layered over HiSep‐1077 (Himedia) solution followed by density gradient centrifugation at 400*g* for 35 min. The PBMCs were counted and plated in 6‐well plates in serum‐free RPMI 1640. After 1 h, media were discarded and PBMCs were washed with PBS to remove any non‐adherent cells. Adhered monocytes were cultured in RPMI 1640 supplemented with 10% heat‐inactivated human serum (Himedia) and 20 ng/mL of M‐CSF (Prospec, Israel). Monocytes were allowed to differentiate in the presence of M‐CSF for 7–10 days, with media changes every 2^nd^ day. Macrophages were then treated with LPS (200 ng/mL) (Sigma‐Aldrich) and IFN‐Ɣ (20 ng/mL) (Prospec) to generate the M1 population while IL‐4 (40 ng/mL) (Prospec) was used to get the M2 population.

### 2.7 Flow Cytometry of Cell Lines and Macrophages

1 × 10^6^ AGS and MKN45 cells were plated in 35‐mm dishes. After infection with *H. pylori* , cells were washed with PBS, trypsinized and stained for CEACAM5 and CEACAM6 expressed on the cell surface. Briefly, cells were stained with the primary antibodies prepared in FACS buffer (0.1% BSA in PBS) for 30 min at 4°C. After washing with FACS buffer, the cells were stained with fluorophore‐conjugated secondary antibodies for 30 min at 4°C in the dark. Cells were washed and acquisition was done using LSRFortessa (BD Biosciences) flow cytometer.

AGS cells stably transfected with the empty vector or *CEACAM6* were cultured on Transwell (Himedia, 0.4 μm pore size) and placed on 6‐wells containing macrophages. After infecting the AGS cells with *H. pylori* at 20 MOI for 48 h, macrophages were detached with Zymefree (Himedia) and stained for surface as well as intracellular markers. Cells were collected, followed by centrifugation and incubated with Fc‐Blocker (Thermo Fisher Scientific) for 15 min. Cells were incubated with fluorophore‐conjugated CD40‐APC, CD64‐PE, and CD206‐APC (Thermo Fisher Scientific) for 30 min in the dark at 4°C. After washing and permeabilisation, cells were incubated with CD68‐FITC (Thermo Fisher Scientific) for 30 min in the dark at 4°C. Cells were washed again and acquisition was performed.

### 2.8 Immunofluorescence Microscopy

1 × 10^5^ AGS cells were plated on coverslips. Staining procedure was followed as described elsewhere [22]. Primary antibody against CEACAM6 was used followed by fluorophore‐conjugated secondary antibody. 4′,6‐diamidino‐2‐ (DAPI; Thermo Fisher Scientific) was used as a counterstain. Images were captured in DMi8 (Leica) confocal microscope. Fluorescence intensity was quantitated using Fiji [23].

Macrophages plated on coverslips were cocultured with empty vector or *CEACAM6* transfected cells with/without *H. pylori* infection on Transwells followed by immunofluorescence staining for iNOS (Thermo Fisher Scientific) and arginase 1 (Thermo Fisher Scientific). Images were captured in Eclipse TiU (Nikon, Tokyo, Japan) epifluorescence microscope. Fluorescence intensity was quantitated using Fiji. Similarly, biopsy tissue samples were immunostained with CD68 (Abcam) and iNOS (Thermo Fisher Scientific) or arginase 1 (Thermo Fisher Scientific) to detect M1 or M2 populations, respectively.

### 2.9 Wound‐Healing Assay

Empty vector or *CEACAM6*‐transfected cells were plated and allowed to grow in a 6‐well plate at full confluency. A 200 μL sterile pipette tip was used to create scratches on these cells. The cells were washed and infected with *H. pylori* at 20 MOI. Images were captured at 0 h and 6 h. Similarly, wounds were created on a confluent layer of AGS, followed by treatment with supernatants derived from uninfected/ *H. pylori* ‐infected empty vector or *CEACAM6* stably transfected AGS cells and images were captured at 0 h and 24 h. All the photomicrographs were taken using an inverted microscope (Nikon).

Difference in area at initial and final time points was measured and expressed as a percentage of wound closure.

### 2.10 Clonogenic Assay

AGS cells stably transfected with the empty vector or *CEACAM6* were plated in a 24‐well plate. After infection with *H. pylori*, cells were trypsinized, counted and 600 cells were plated in 35‐mm dish. After 10 days, colonies were washed in PBS and subjected to 4% paraformaldehyde fixation. Crystal violet (0.5%) was used for staining the colonies. Images of the plates were captured, and colonies were counted.

Equal number of AGS cells was treated with supernatants collected from the empty vector or *CEACAM6*‐expressing AGS cells for 24 h. After treatment, 600 cells were plated in 35‐mm dish. Again, colonies were allowed to develop, and the rest of the procedure was followed as mentioned above.

### 2.11 Population Doubling Assay

0.1 × 10^6^ empty vector or *CEACAM6*‐overexpressing AGS cells were plated in a 24‐well plate. After infection with *H. pylori* , cells were washed, trypsinized and 10^4^ cells were plated in a 24‐well plate. At days 2, 3 and 4, cells were trypsinized and counted. The following formula was used for evaluating population doubling:

$\mathrm{PD}=\left[\log\left(\mathrm{Nh}\right)-\log\left(\mathrm{Ns}\right)\right]/\log\;2.$

Ns and Nh represent the number of seeded and harvested cells, respectively. After the addition of the PD values obtained for each day, the cumulative population doubling (cPD) was enumerated [22]. A similar procedure was followed for evaluating the effect of supernatants derived from the empty vector or *CEACAM6*‐overexpressing AGS cells on AGS cells.

### 2.12 Transwell Invasion Assay

Equal numbers of the empty vector or *CEACAM6*‐expressing AGS cells were cultured in a 24‐well plate. After infection, the cells were washed, trypsinized and replated on Transwells (Himedia, 8 μm pore size) coated with matrigel (Sigma‐Aldrich). After 24 h, the Transwells were processed as described previously [19, 24] and stained with crystal violet (0.5%). Bright field images were captured using an inverted microscope (Nikon) and the cells were counted.

Equal numbers of AGS cells were treated with supernatants derived from the empty vector or *CEACAM6*‐expressing AGS cells for 24 h. After treatment, an invasion assay was performed as described above.

### 2.13 Statistical Analysis

Statistical analyses were conducted using GraphPad Prism 9.5.1 (GraphPad, CA, USA). All the experiments were repeated at least thrice. Data were expressed as mean ± sem. Statistical significance was established by Student's *t*‐test or two‐way ANOVA. Post‐hoc analysis was executed by Tukey's test.

## 3 Results

### 3.1 CEACAMs are Upregulated in *H. pylori* ‐Infected GCCs

**FIGURE 1:**
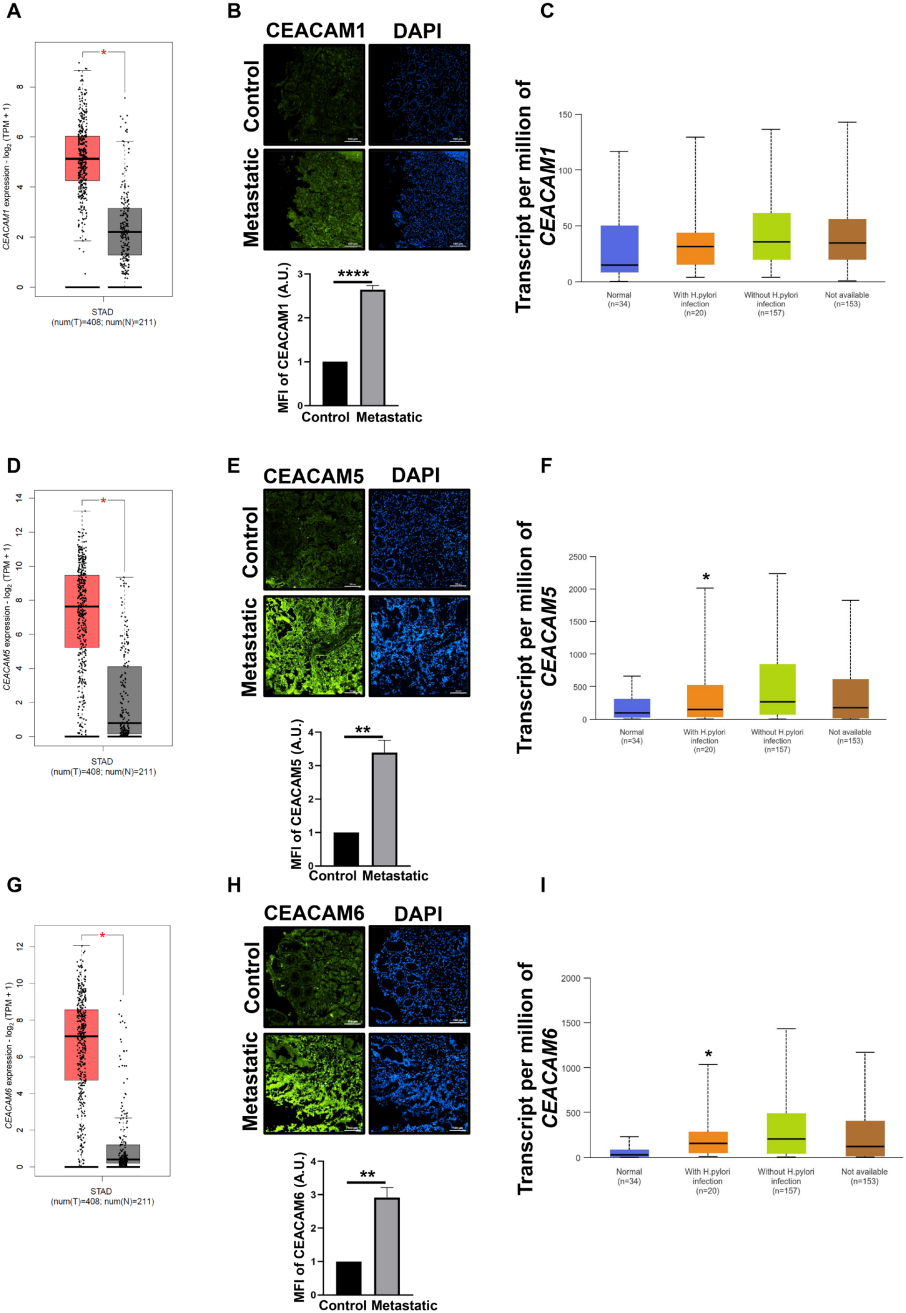
CEACAMs are upregulated in GC patients. (A, D, G) The plots show transcripts per million (TPM) levels of *CEACAM1*, *CEACAM5* and *CEACAM6* in normal versus stomach cancer tissue in GEPIA database. (B, E, H) Immunofluorescence images show levels of CEACAM1 (green), CEACAM5 (green) and CEACAM6 (green) in metastatic patients versus paired control (*n* = 3). DAPI (blue) stains the nuclei. Images are captured with 20× objective. Scale bar = 100. Corresponding bar graphs show fold change in mean fluorescent intensity of CEACAM1, CEACAM5 and CEACAM6 levels in metastatic patients relative to paired controls. Data are represented as mean ± sem. Student's *t*‐test is performed to determine the statistical significance. *p < 0.05, **p < 0.01, ****p < 0.0001. (C, F, I) UALCAN analysis of TCGA STAD show transcripts per million (TPM) levels of *CEACAM1*, *CEACAM5* and *CEACAM6* in healthy individuals and patients with or without *H. pylori* infection.

Analysis of the STAD database in the GEPIA revealed that among the 4644 differentially‐expressed genes, *CEACAM5* and *CEACAM6* are the top two genes, while *CEACAM1* remains among the top 250 (http://gepia.cancer‐pku.cn/detail.php?clicktag=degenes, at keeping Log_2_FC cutoff: 1, q‐value cutoff set at: 0.01, differential method: ANOVA and chromosomal distribution: over‐expressed). Box‐plot analyses of the STAD database in GEPIA also showed significant upregulation of *CEACAM1*, *CEACAM5* and *CEACAM6* in GC (Figure 1A,D,G). We validated these results by immunofluorescence microscopy of these CEACAMs in adenocarcinoma, metastatic human GC biopsy tissue samples and their paired controls. Results confirmed that CEACAM1, CEACAM5 and CEACAM6 were significantly high in metastatic tissue samples (*p* < 0.0001, *p* < 0.01 and *p* < 0.01, respectively) (Figure 1B,E,H). However, changes in levels of CEACAMs in gastric adenocarcinoma were not significant (Figure S1A). Within the UALCAN database, stage‐specific analysis of transcripts of *CEACAM1*, *CEACAM5* and *CEACAM6* from TCGA STAD showed significantly high expression in stage 3 and stage 4 of GC patients (Figure S1B). To understand the association of *H. pylori* infection with *CEACAM* expression, we retrieved *H. pylori* ‐specific information about patients from the UALCAN database. Here, levels of *CEACAM5* and *CEACAM6* were significantly elevated in samples infected with *H. pylori* (Figure 1F,I, respectively). Interestingly, no significant change in the expression of *CEACAM1* was observed between normal and *H. pylori‐*infected samples (Figure 1C).

**FIGURE 2:**
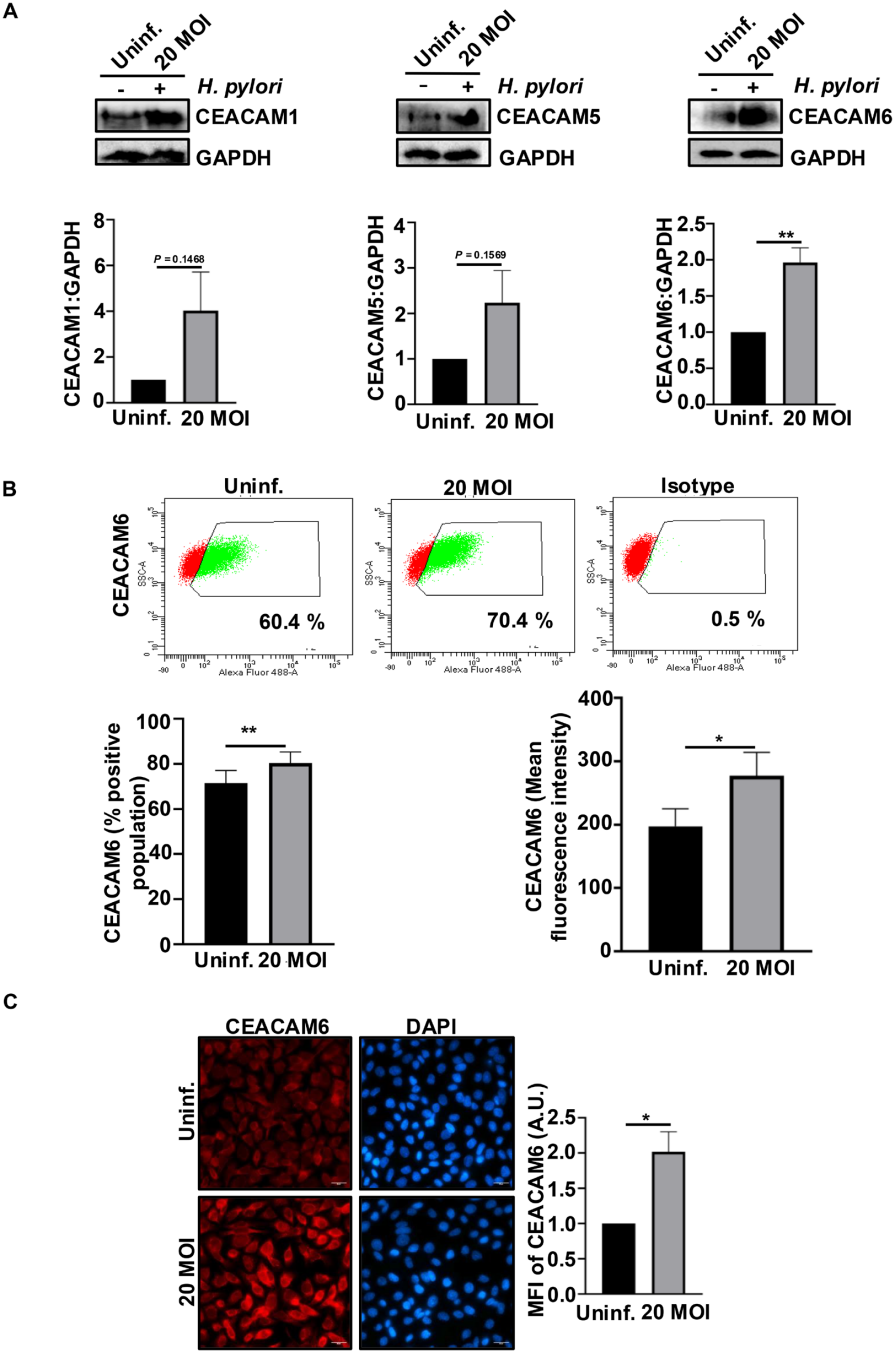
*H. pylori* upregulates CEACAM6 in GCCs. (A) Western blotting of whole cell lysates showing CEACAM1, CEACAM5 and CEACAM6 level post *H. pylori* infection. GAPDH is used as a loading control. Bar graphs show fold change in expression of CEACAMs relative to that of uninfected cells. (B) Representative dot plots (*n* = 3) of flow cytometry analyses show surface levels of CEACAM6 upon infection with *H. pylori* at 20 MOI for 24 h. Graphs show percent positive population for CEACAM6 and mean fluorescence intensity of CEACAM6 MKN45. (C) Confocal micrographs showing CEACAM6 (red) level within cells after infection. Nuclei are stained with DAPI (blue). Images are captured using 63× objective. Scales represent 20 μm. Graphs represent the fold change in mean fluorescence intensity of CEACAMs relative to that of uninfected cells in arbitrary units (A.U.). Data are represented as mean ± sem. Student's *t*‐test is performed to determine the statistical significance. *p < 0.05, **p < 0.01. Uninf., uninfected.

To validate the effect of *H. pylori* in modulating CEACAMs, we performed in vitro analysis using AGS and MKN45 cell lines. CEACAM1, CEACAM5, and CEACAM6 protein levels were assessed after infecting AGS with *H. pylori cag* (+) strain‐26695 for 24 h. Immunoblotting was performed to assess the status of CEACAMs in whole cell lysates of infected AGS and MKN45 cells. Only CEACAM6 was significantly elevated in AGS and MKN45 cells upon infection (Figure 2A; Figure S2A). Flow cytometry data revealed the surface expression of CEACAM6. The CEACAM6‐positive population and mean fluorescence intensity of CEACAM6 increased significantly in *H. pylori*‐infected AGS compared to uninfected cells (Figure 2B). MKN45 also exhibited significant upregulation of the CEACAM6‐positive population and mean fluorescence intensity of CEACAM6 after infection (Figure S2B). In a study, P12 and G27 strains of *H. pylori* showed an increasing trend in *CEACAM* expression, though the results were not statistically significant [5]. We further performed immunofluorescence microscopy and a significant increase in the CEACAM6 protein level was noticed in infected AGS cells compared to uninfected AGS cells (Figure 2C). Since MKN45 cells grow in clusters, we didn't use these cells to generate microscopy results. Based on bioinformatics analysis and our findings with *H. pylori* at 20 MOI, CEACAM6 exhibited prominence. Recently, CEACAM6 was found to gain importance as a biomarker and a prognostic marker for GC aggressiveness [25, 26]. Therefore, the role of CEACAM6 in the context of *H. pylori* ‐mediated GC was further explored in this study.

### 3.2 *CEACAM6* ‐Overexpressing Epithelial Cells Promote Cell Proliferation, Migration and Invasion

**FIGURE 3:**
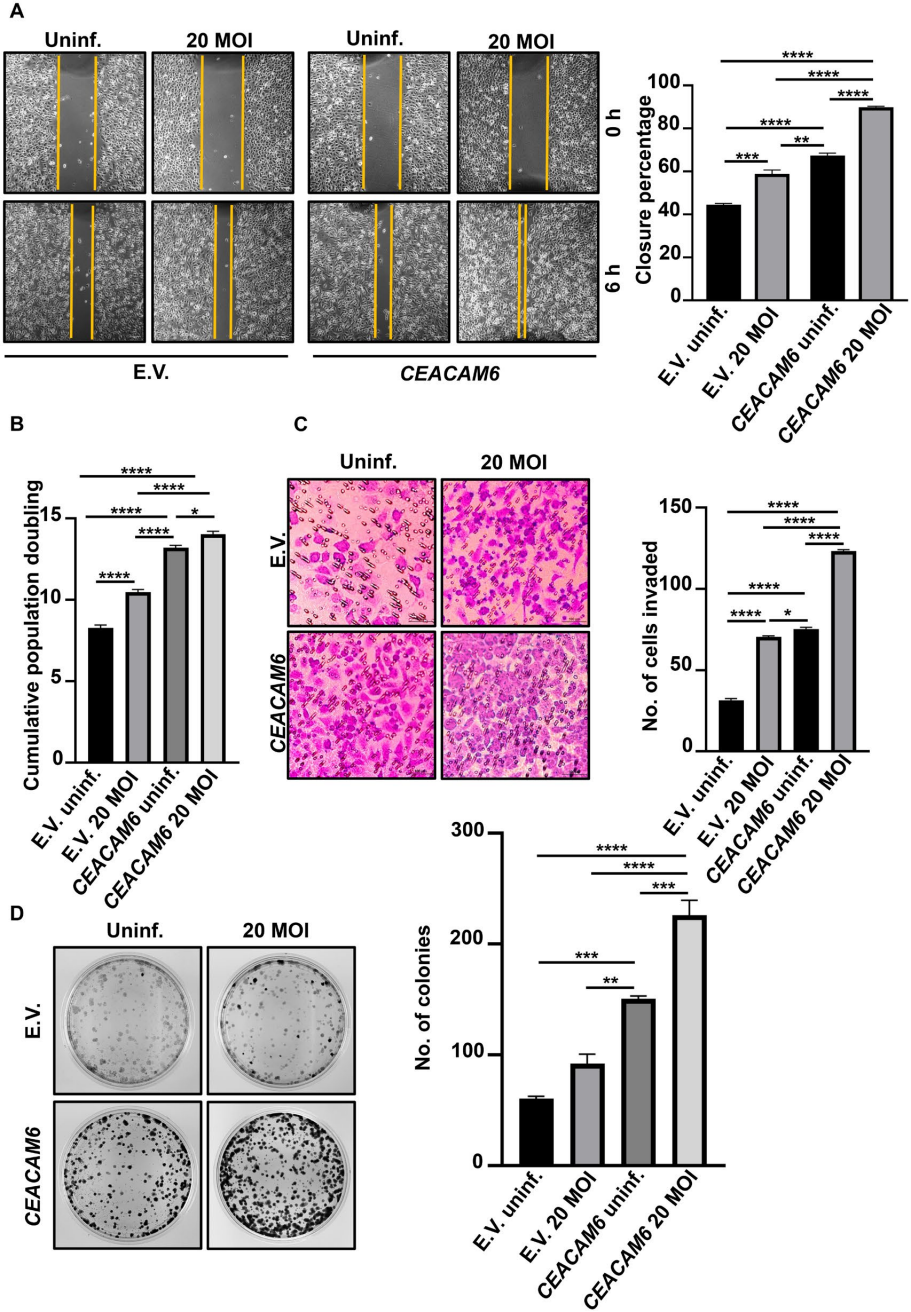
CEACAM6 promotes aggressiveness in AGS. (A) Brightfield microscopy of wound closure by AGS cells stably expressing the empty vector or *CEACAM6* with or without *H. pylori* infection for 6 h. Images are captured using a 10× objective. Scale bars = 100 μm. Graph represents the percentage of wound closure. (B) Graph showing the cumulative population doubling of AGS cells stably expressing the empty vector or *CEACAM6* with or without *H. pylori* infection. (C) Brightfield micrographs showing the invasive potential of AGS cells transfected with the empty vector or *CEACAM6* with or without *H. pylori* infection after the matrigel invasion assay. A 20× objective is used to capture images. Scale bars = 100 μm. Graph represents the number of cells invaded. (D) Clonogenic assay depicts the colony‐forming abilities of AGS cells stably expressing the empty vector or CEACAM6 with or without *H. pylori* infection. The graph indicates the number of colonies. Two‐way ANOVA followed by Tukey's post hoc analysis is performed to determine statistical significance. All data are expressed as mean ± sem (*n* = 3). *p < 0.05, **p < 0.01, ***p < 0.001, ****p < 0.0001. E.V., empty vector; Uninf., uninfected.

CEACAM6 overexpression has been correlated with adverse clinical outcomes in GC—it is a contributing factor in tumour aggressiveness and confers resistance against therapy [25, 27]. However, no report exists on the influence of CEACAM6 on *H. pylori* ‐mediated GC pathogenesis [28]. To assess the role of CEACAM6 in modulating cell motility, AGS cells were transfected with the empty vector or *CEACAM6* plasmid to generate stable cells. These cells were then infected with *H. pylori* at 20 MOI, and a wound‐closure assay was performed. Significantly increased migration of *CEACAM6*‐overexpressing cells was noticed compared to empty vector‐expressing cells (*p* < 0.0001) (Figure 3A). This effect was further enhanced after infection with *H. pylori* . To assess the effect of *CEACAM6* overexpression on cell proliferation, the population doubling assay was performed. *CEACAM6*‐overexpressing infected cells showed a significant increase in cell number compared to the other experimental groups (Figure 3B). Matrigel‐based Transwell invasion assay was carried out to measure the directed movement of the *CEACAM6*‐overexpressing cells with/without *H. pylori* infection towards a chemoattractant (RPMI 1640 supplemented with 20% FBS). CEACAM6 significantly promoted invasion compared to uninfected or infected empty vector‐overexpressing cells, which was further enhanced by *H. pylori* infection (Figure 3C). The role of CEACAM6 in inducing the colony‐forming ability of AGS cells was assessed by clonogenic assay, which is a standard method to estimate the growth of a cancer cell to form a colony. *CEACAM6*‐stably expressing cells showed significantly high colony‐forming ability as compared to the empty vector cells, which further increased after *H. pylori* infection (Figure 3D).

### 3.3 Supernatant from *H. pylori* ‐Infected *CEACAM6* ‐Overexpressing GCCs Promote Cell Proliferation, Migration and Invasion of Recipient GCCs

**FIGURE 4:**
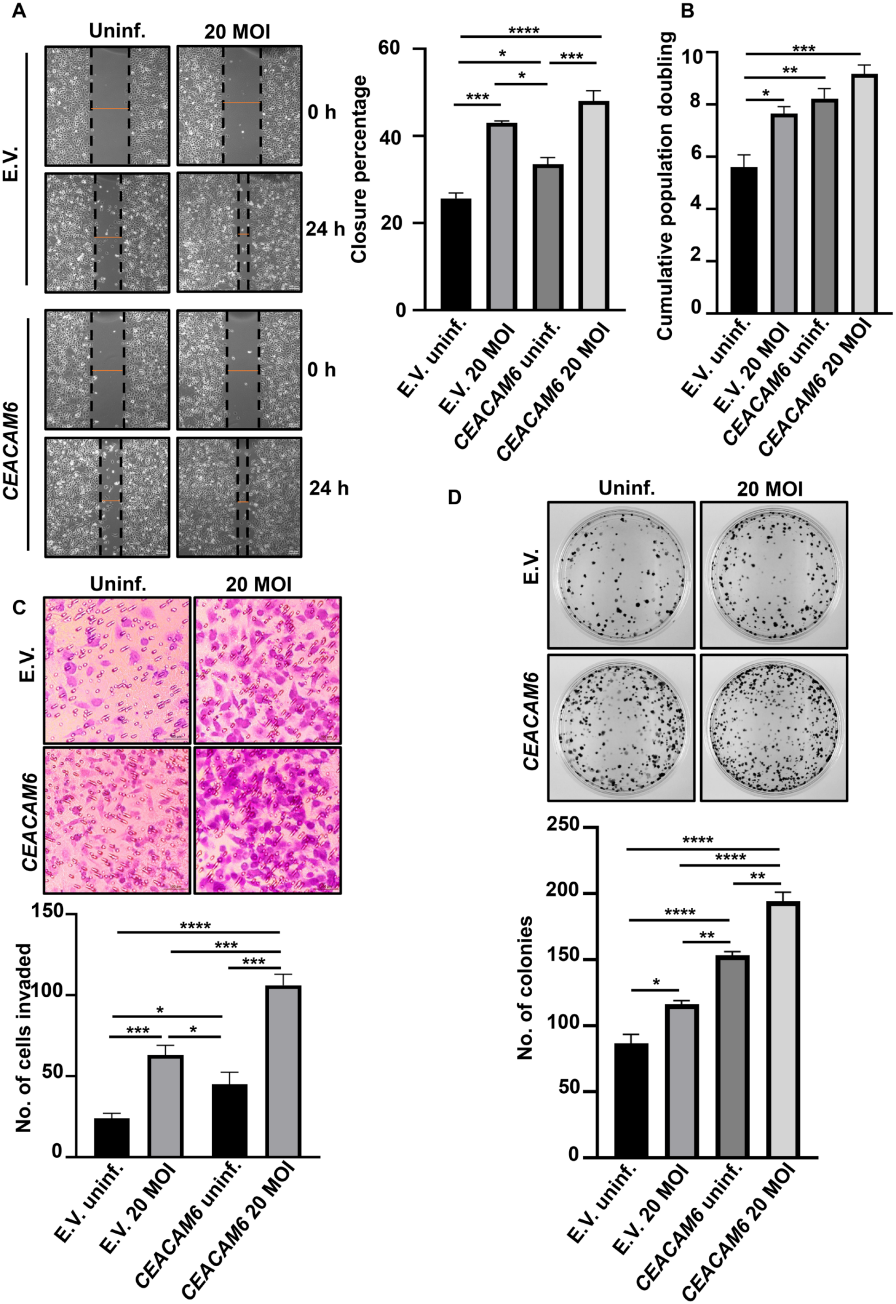
Supernatant derived from *CEACAM6‐*overexpressing cells contributes to the aggressiveness of AGS. (A) Representative images show the effect of supernatants derived from empty vector or *CEACAM6‐*stably transfected cells with or without *H. pylori* infection on wound closure of AGS cells. Graph shows the percentage wound closure after 24 h. Images are captured using a 10× objective. Scale bars represent 100 μm. (B) Graph represents the cumulative population doubling of AGS upon treatment with supernatants derived from empty vector or *CEACAM6‐*stably transfected cells with or without *H. pylori* infection. (C) Invasion assay shows the invasiveness of AGS cells treated with supernatants derived from empty vector or *CEACAM6‐*stably transfected cells with or without *H. pylori* infection. A 20× objective is used to capture images. Scale bars represent 100 μm. (D) Photographs of clonogenic assay reveal the ability to form colonies of AGS cells when treated with supernatants derived from the empty vector or *CEACAM6‐*stably transfected cells with or without *H. pylori* infection. Graph shows the number of colonies after counting. Two‐way ANOVA followed by Tukey's post hoc analysis is performed to determine statistical significance. All data are represented as mean ± sem (*n* = 3). *p < 0.05, **p < 0.01, ***p < 0.001, ****p < 0.0001. E.V., empty vector; Uninf., uninfected.

Neoplastic epithelial cells can induce the transformation of the adjacent non‐transformed epithelial cells in the TME [29]. Therefore, we explored the impact of the supernatant derived from *CEACAM6*‐expressing GCCs on the non‐expressing GCCs. Treated cells were used in wound‐healing and population doubling assays to assess the migration and proliferation abilities of AGS cells. Supernatants from *CEACAM6*‐overexpressing cells enhanced the migration ability of AGS cells over that of empty vector‐overexpressing cells (Figure 4A). Data also revealed that the supernatant from infected *CEACAM6*‐overexpressing cells significantly enhanced the migration ability of the recipient cells among all treatment groups. Similarly, the population doubling assay using AGS cells revealed that supernatants from *CEACAM6*‐expressing infected cells significantly induced proliferation of the recipient cells (Figure 4B). Furthermore, the matrigel‐based Transwell invasion assay demonstrated that supernatants derived from infected empty vector‐transfected AGS cells promoted significant invasion of AGS cells over that of the uninfected setup. Additionally, AGS treated with supernatants derived from infected *CEACAM6*‐overexpressing cells exhibited significantly high invasion compared to that of all other experimental conditions (Figure 4C). Next, we performed a clonogenic assay to assess the effectiveness of these supernatants on the survival and proliferative ability of AGS cells. The number of colonies significantly increased in AGS cells treated with supernatants derived from infected *CEACAM6*‐expressing cells, which also reflected the weak contact inhibition property of these cells (Figure 4D).

### 3.4 Infected GCCs Regulate Macrophage Polarisation by CEACAM6

**FIGURE 5:**
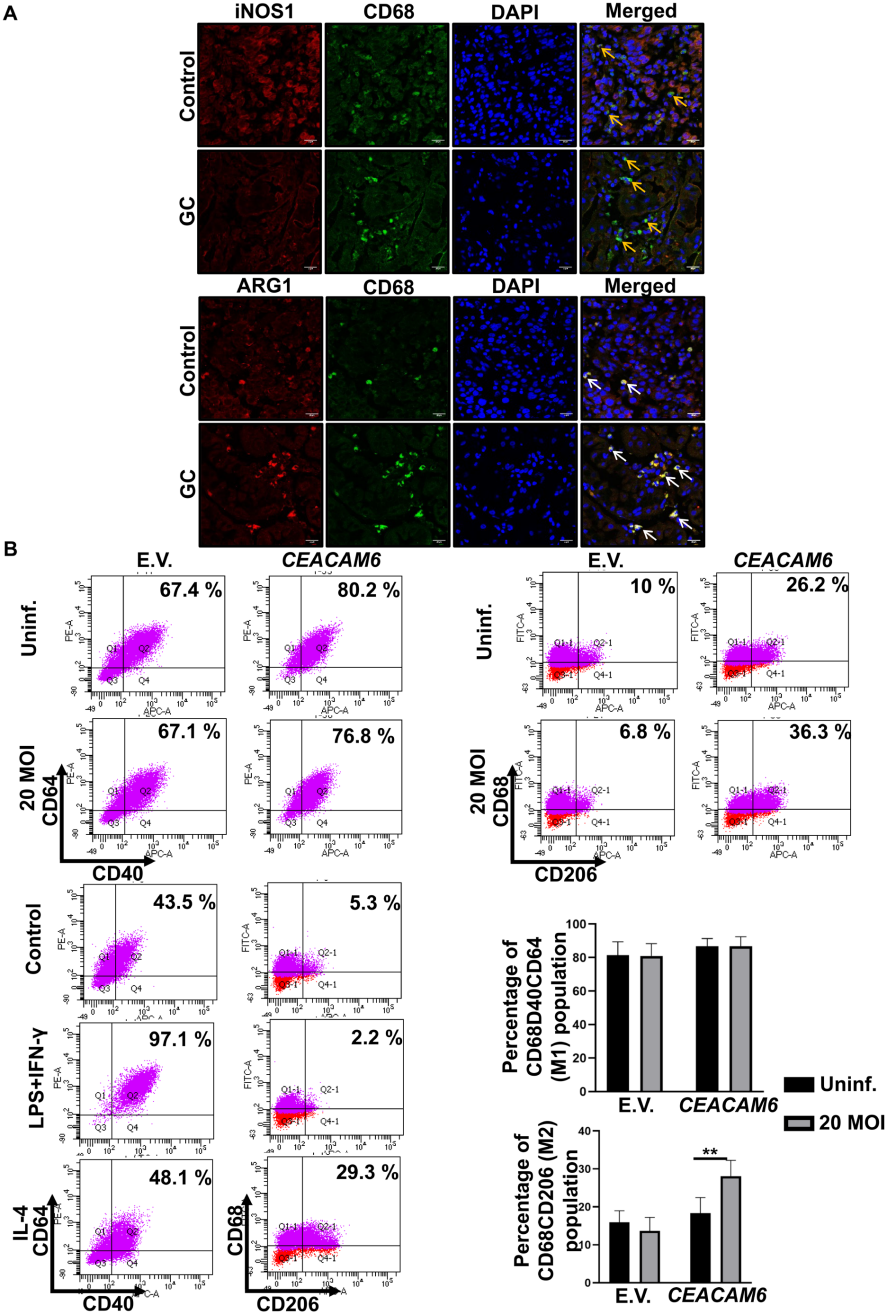
Epithelial CEACAM6 drives M2 polarisation of the macrophages. (A) Confocal microscopy images show CD68 (green) with iNOS (red) or arginase 1 (ARG1) (red) in biopsy tissue obtained from antral region of gastric adenocarcinoma patient (*n* = 3). Nuclei are stained with DAPI (blue). 63× objective is used. Scale bar = 20 μm. (B) Representative dot plots show CD68^+^CD40^+^CDC64^+^(M1) and CD68^+^CD206^+^(M2) population of macrophages after flow cytometry analysis of macrophages cocultured with *CEACAM6*‐overexpressing AGS cells. LPS (100 ng/mL) and IFN‐γ (20 ng/mL) or IL‐4 (40 ng/mL) treated macrophages serve as positive controls for M1 or M2 polarised macrophages. Bar graphs represent M1 and M2 population change in macrophages after coculture. Student's *t*‐test is conducted to determine statistical significance. **p < 0.01. E.V., empty vector; Uninf., uninfected.

In *H. pylori* infection, gastritis develops due to the accumulation of mononuclear cells in the gastric mucosa [30]. Macrophage infiltration is a significant event within the TME that can influence tumour growth [31]. Circulating monocytes are recruited to the tumour and under the influence of colony‐stimulating factor‐1, get differentiated into macrophages [32]. Within the TME, these macrophages differentiate into M1 or M2 phenotypes. M1 macrophages, being pro‐inflammatory, inhibit tumour progression, while anti‐inflammatory M2 macrophages actively promote tumour progression and metastasis. To assess the infiltration and polarisation status of macrophages in GC, biopsy tissues obtained from gastric adenocarcinoma patients were immunostained with CD68 (pan‐macrophage marker) and iNOS (M1 marker) or arginase 1 (M2 marker). The number of macrophages was high in adenocarcinoma tissues compared to the paired controls, as evident from immunofluorescence staining of CD68 (Figure 5A). Moreover, M2 macrophages (CD68 and arginase 1‐immunofluorescence) were significantly high in adenocarcinoma compared to their paired controls. In contrast, the number of macrophages stained for CD68 and iNOS was much less in GC in comparison to the paired control (Figure 5A). To further explore the overall influence of GCCs‐expressing CEACAM6 in modulating macrophage polarisation, macrophages on 6‐well plates were cocultured with *CEACAM6*‐overexpressing AGS cells cultured on Transwell inserts. Following this, empty vector and *CEACAM6* stably‐transfected cells were infected with *H. pylori* at 20 MOI for 48 h. After treatment, macrophages were stained with CD40, CD64 and CD68 markers to assess the M1 phenotype. CD68 and CD206 were used to assess the M2 phenotype. Flow cytometry was performed by gating on the CD68 population corresponding to macrophages. Then, the CD40^+^CD64^+^ population and CD206^+^ population were assessed. Results showed that the CD68^+^CD40^+^CD64^+^ macrophage population remained unchanged when cocultured with empty vector and *CEACAM6* stably‐transfected cells irrespective of *H. pylori* infection. Interestingly, macrophages cocultured with *CEACAM6* stably‐transfected and *H. pylori* ‐infected cells showed a significantly high CD68^+^CD206^+^ population compared to the macrophages cocultured with *CEACAM6* stably‐transfected uninfected cells (Figure 5B). We also performed immunofluorescence microscopy of macrophages after coculturing with GCCs and staining with iNOS (M1 marker) as well as arginase 1 (M2 marker) and measured fluorescence levels (Figure S3A,B). Once again, macrophages cocultured with *H. pylori* ‐infected stably‐expressing *CEACAM6* cells stained significantly more for arginase 1. It was crucial to identify the soluble factors released by epithelial cells that can modulate macrophage polarisation towards the M2 phenotype. For that, a cytokine array analysis of supernatants derived from the empty vector or *CEACAM6*‐expressing cells with/without infection was performed (Figure S4). *CEACAM6*‐expressing cells with *H. pylori* infection noticeably increased IL‐8, soluble TNFR1 and RANTES when compared with other groups. IL‐8 and RANTES favour M2 macrophage polarisation [33, 34, 35]. TNF‐α promotes M1 polarisation, soluble TNFR1 binds free TNF‐α and therefore downregulates inflammatory responses, thus contributing to M2 polarisation of macrophages [36, 37]. Among all groups, infected *CEACAM6*‐expressing cells showed the lowest levels of IL‐16, which plays a role in promoting M1 polarisation of macrophages [38].

Collectively, our data confirmed that *CEACAM6*‐expressing *H. pylori* ‐infected GCCs promoted tumorigenic responses. Moreover, soluble mediators released by these cells imparted proliferative and invasive attributes to the uninfected GCCs and promoted M2 polarisation of macrophages.

## 4 Discussion

This study explored the effect of *H. pylori* infection in modulating CEACAMs in GCCs. Our data revealed that among CEACAM1, CEACAM5 and CEACAM6, only CEACAM6 was significantly upregulated by *H. pylori* and potentiated cell migration, proliferation as well as invasiveness of GCCs. Interestingly, *CEACAM6*‐expressing infected cells imparted a paracrine effect on uninfected GCCs and resulted in similar increases in the latter group of cells. Further, soluble mediators released by *CEACAM6*‐overexpressing cells increased M2 macrophage population. Thus, this study confirmed that *H. pylori* ‐mediated upregulation of CEACAM6 could enhance the tumorigenic potential of GCCs and promote M2 polarisation of macrophages.

The upregulation of specific CEACAM members, notably CEACAM1, CEACAM5 and CEACAM6, has been observed in several malignancies, including GC. However, this study found significant upregulation of CEACAM6 by *H. pylori* , enhancing the tumorigenic potential of GCCs. The supernatant derived from *CEACAM6*‐expressing GCCs stimulates the angiogenic potential of HUVEC cells [26]. This observation underscores the fact that soluble factors released by *CEACAM6*‐expressing cells also play a role in tumor progression. Our results corroborated these findings as we observed supernatants derived from *CEACAM6*‐expressing cells enhanced cellular mobility, proliferation and invasiveness in GCCs. The effect was prominent with the supernatant derived from *H. pylori‐*infected *CEACAM6*‐overexpressing cells.

CEACAMs also modulate the immune system to favor cancer promotion. For example, tumor cells employed CEACAM1 to inhibit the activity of NK cells and T cells to escape immune surveillance [39]. Stern et al. showed that CEACAM5 nullified the anti‐cancer activity of NK cells [40]. Macrophages, the most abundant immune cells in the TME, significantly influence tumor progression. A recent article from our group showed that supernatants derived from *CEACAM6*‐expressing hypoxic cells could promote the M2 polarisation of macrophages [41]. *H. pylori* infection was also linked with M2 polarisation of macrophages [42]. Our study for the first time linked M2 polarisation of macrophages with CEACAM6 upregulation in GCCs caused by *H. pylori* infection. The outcome of infection with *H. pylori* is heavily dependent on bacterial load. Variation in MOI may yield contrasting effects. Existing reports indicated that infection of macrophages with *H. pylori* at low MOI induced both M1 and M2 phenotypes. However, high MOI suppressed the M2 phenotype [43]. Abundant M2 macrophages were linked to immunosuppression, poor overall survivability [44], pro‐angiogenic effects [45] and increased invasiveness in GC [46]. Further studies are required to unravel the therapeutic intervention potential of CEACAM6‐mediated M2 polarisation in the context of GC.

Even though earlier studies showed CEACAM upregulation after *H. pylori* infection in GCCs, those focused on assessing transcript levels of CEACAMs [5, 47]. This study found a significant upregulation of CEACAM6 protein post‐infection. However, cells were infected for a longer duration than had been the case for other studies. Variabilities in the *H. pylori* ‐driven CEACAM1, CEACAM5 and CEACAM6 levels within cell lines could be ascribed to the duration and MOI. Reports indicated that CEACAM glycosylation is important for their stability as well as function [48]. Therefore, the discrepancies in the CEACAM levels within cell lines could be associated with the glycosylation level of these proteins. Future studies should focus on the level of glycosylation of CEACAMs and subsequent changes in expression in response to *H. pylori* infection. An in‐depth analysis of factors released by *CEACAM6*‐expressing cells needs to be carried out to identify crucial factors that can modulate the tumorigenic potential of GCCs as well as the polarisation of macrophages. Homotypic interaction among CEACAMs as well as heterotypic interactions of CEACAMs with other adhesion proteins in GCCs could alter the *H. pylori* ‐mediated GC pathogenesis, which warrants further exploration.

In summary, this study found that CEACAM6 was upregulated in *H. pylori* ‐infected GCCs. Elevated CEACAM6 levels in GCCs ensured protumorigenic responses of these cells resulting in proliferative and invasive effects even from the GCCs not infected with *H. pylori* . Likewise, soluble mediators released by *CEACAM6*‐expressing infected GCCs potentiated M2 polarisation of macrophages. Thus, these findings advanced our knowledge regarding the importance of CEACAM6 in *H. pylori* infection and GC pathogenesis.

## Author Contributions

**Debashish Chakraborty:** data curation (lead), formal analysis (lead), investigation (lead), writing – original draft (lead), writing – review and editing (lead). **Indrajit Poirah:** writing – review and editing (supporting). **Supriya Samal:** writing – review and editing (supporting). **Smaran Banerjee:** writing – review and editing (supporting). **Aranya Pal:** writing – review and editing (supporting). **Chandan Mahish:** data curation (supporting). **Subhasis Chattopadhyay:** formal analysis (supporting). **Girija Nandini Kanungo:** resources (supporting). **Pusparaj Samantasinhar:** resources (supporting). **Gautam Nath:** resources (supporting). **Niranjan Rout:** resources (supporting). **Shivaram Prasad Singh:** resources (supporting). **Asima Bhattacharyya:** conceptualization (lead), formal analysis (lead), investigation (lead), methodology (lead), project administration (lead), resources (lead), supervision (lead), writing – original draft (lead), writing – review and editing (lead).

## Ethics Statement

Human tissue was collected with prior informed consent from the patients and was approved by the Institutional Ethics Committee for Human Research, National Institute of Science Education and Research (NISER) (protocol No. NISER/IEC/2018–01).

## Conflicts of Interest

The authors declare no conflicts of interest.

## Supporting information


Figures S1–S4.

## Data Availability

The datasets used and/or analysed during the current study are available from the corresponding author on reasonable request.
