# Supplementary material for: Protumorigenic Responses of CEACAM6 in Helicobacter pylori‐Infected Gastric Cancer Cells
Source: J Cell Mol Med. 2026 Jan 28;30(2):e70869. doi: 10.1111/jcmm.70869 (PMC12848906; doi:10.1111/jcmm.70869)
Supplement: Supplementary file 1 — Figures S1–S4. [file JCMM-30-e70869-s001.pdf]

Supplementary information

Protumorigenic responses of CEACAM6 in *Helicobacter pylori*-infected gastric cancer cells

Debashish Chakraborty[1], Indrajit Poirah[1], Supriya Samal[1], Smaran Banerjee[1], Aranya Pal[1], Chandan Mahish[1], Subhasis Chattopadhyay[1], Girija Nandini Kanungo[2], Pusparaj Samantasinhar[3], Gautam Nath[4], Niranjan Rout[5], Shivaram Prasad Singh[5], Asima Bhattacharyya[1,6*]

Affiliations:[1]School of Biological Sciences, National Institute of Science Education and Research (NISER) Bhubaneswar, An OCC of Homi Bhabha National Institute, P.O. Bhimpur-Padanpur, Via Jatni, Dist. Khurda 752050, Odisha, India; [2]Immuno Haematology and Blood Transfusion, IMS and SUM hospital, Kalinganagar, Bhubaneswar 751003, Odisha, India; [3]Forensic Medicine and Toxicology, IMS and SUM hospital, Kalinganagar, Bhubaneswar 751003, Odisha, India; [4]Department of Gastroenterology, Acharya Harihar Post Graduate Institute of Cancer, Cuttack 753007, Odisha, India; [5]Digestive Diseases Centre, Beam Diagnostics Building, Bajrakabati Road (Shanti Nagar), Cuttack 753001, Odisha, India; [6]Centre for Interdisciplinary Sciences (CIS), NISER, An OCC of Homi Bhabha National Institute, P.O. Bhimpur-Padanpur, Via Jatni, Khurda 752050, Odisha, India.

Correspondence: School of Biological Sciences, National Institute of Science Education and Research (NISER) Bhubaneswar, An OCC of Homi Bhabha National Institute, P.O. Bhimpur-Padanpur, Via Jatni, Khurda, 752050, Odisha, India. Tel: +91-674-2494210, Email: asima@niser.ac.in

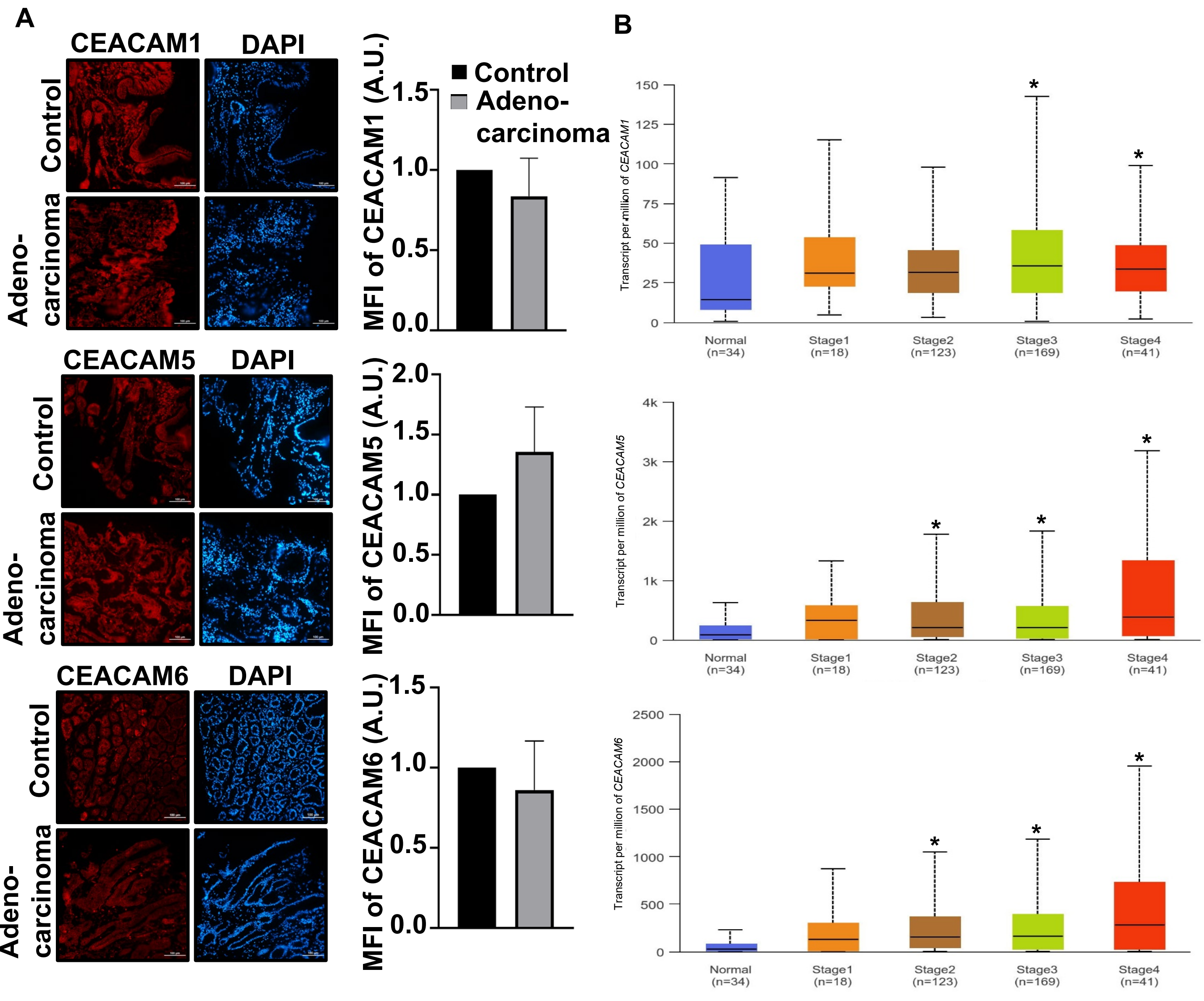

Fig. S1. CEACAMs are upregulated in GC patients. (**A**) Immunofluorescence images show levels of CEACAM1 (red), CEACAM5 (red) and CEACAM6 (red) in adenocarcinoma patients vs paired control (n=3). DAPI (blue) is used to stain the nuclei. Images are captured with 20X objective. Bar graphs represent fold change in mean fluorescent intensity of CEACAM1, CEACAM5 and CEACAM6 levels in adenocarcinoma patients relative to paired controls. Scale bar=100 μm. Data are expressed as mean ± sem **(B)** Box plots depict levels of *CEACAM1*, *CEACAM5* and *CEACAM6* as transcripts per million in different stages of cancer after analysis in UALCAN.

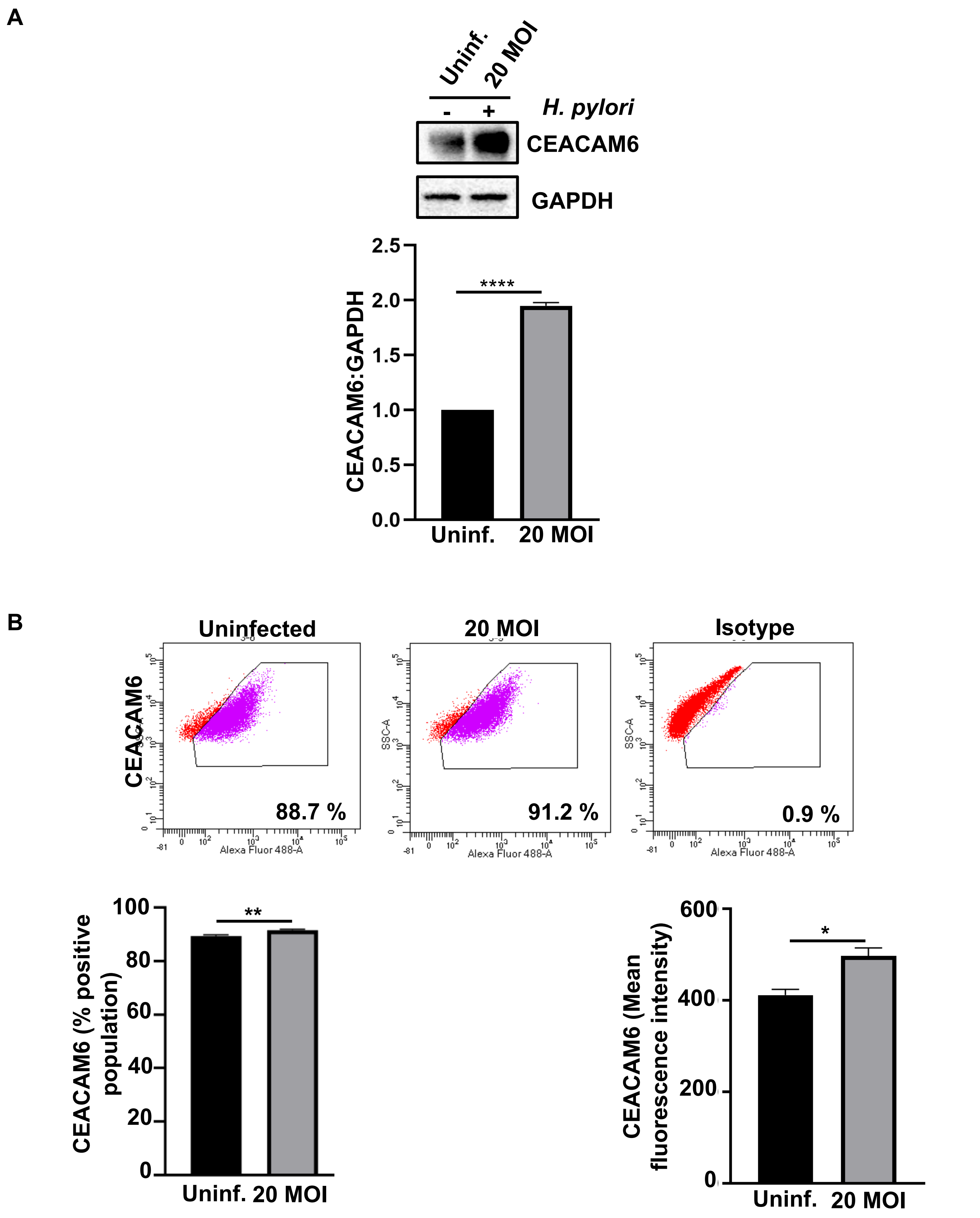

Fig. S2. *H. pylori* upregulates CEACAM6 in GCCs. (**A**) Representative immunoblots show expression of CEACAM6 in MKN45 cells after *H. pylori* infection. GAPDH serves as loading control. Graph indicates fold change in the expression of CEACAM6 in uninfected/infected cells. (**B**) Dot plots show surface expression of CEACAM6 in MKN45 cells. Bar graph represents percent positive population for CEACAM6 and mean fluorescence intensity of CEACAM6 MKN45. Data are represented as mean ± sem. Student's t-test is performed to show statistical significance. $^{*}p < 0.05$, $^{**}p < 0.01$, $^{****}p < 0.0001$. Uninf., uninfected.

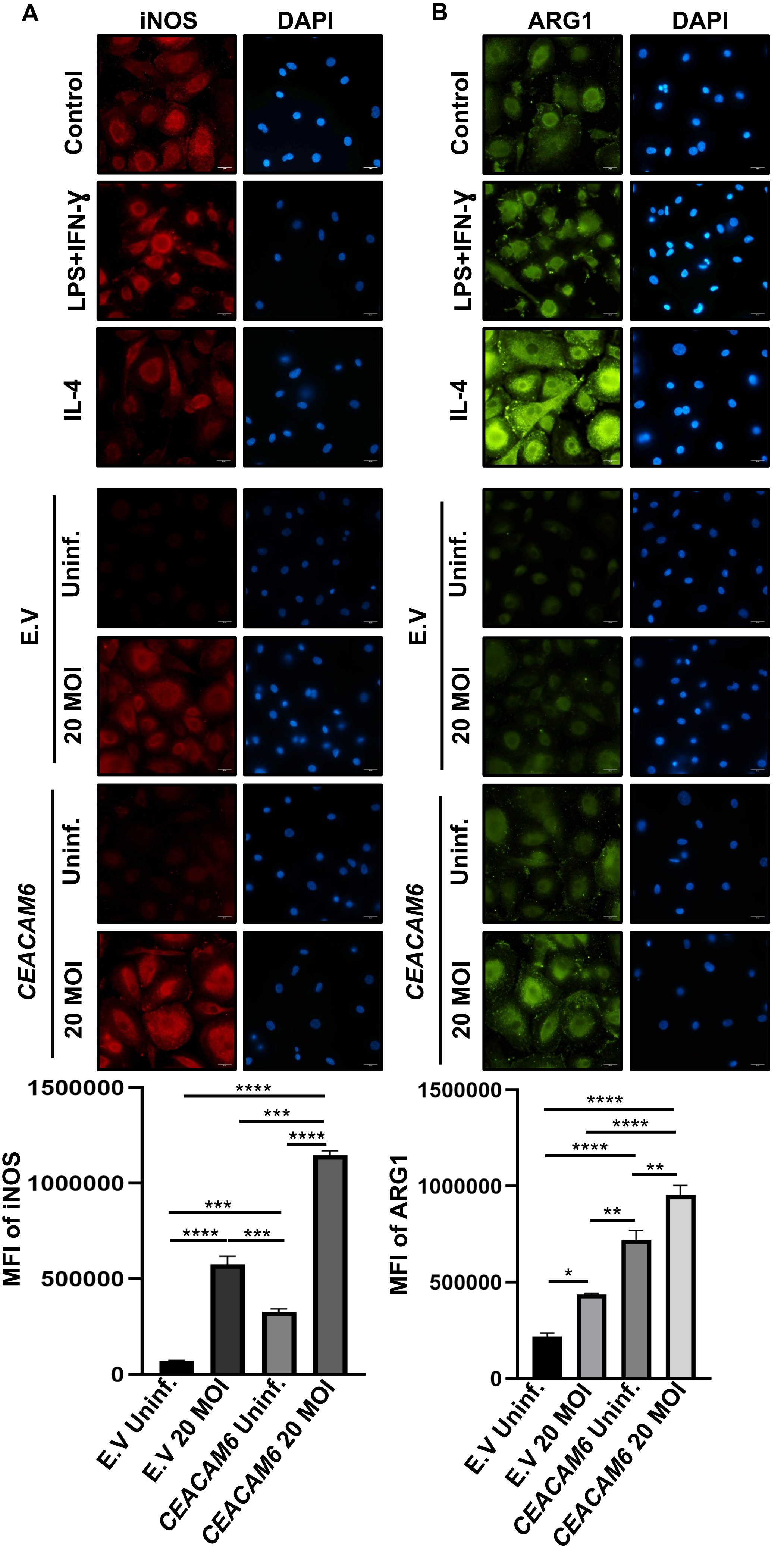

Fig. S3. Epithelial CEACAM6 promotes M2 polarisation of macrophages.
(**A**) Representative immunofluorescence images (n=3) shows inducible nitric oxide synthase (iNOS) (red) and arginase 1 (ARG1) (green) levels in macrophages cocultured with *CEACAM6*-expressing AGS cells. Nuclei are stained with DAPI (blue). Objective used=60X. Scale bar=20 µm. Mean fluorescence intensity of iNOS and ARG1 are plotted as bar graphs. LPS (100 ng/ml) and IFN-γ (20 ng/ml) or IL-4 (40 ng/ml) treated macrophages serve as positive controls for M1 or M2 macrophages, respectively. Two-way ANOVA followed by Tukey's post-hoc analysis is performed to determine statistical significance. All data are expressed as mean ± sem (n=3). $^{*}p < 0.05$, $^{**}p < 0.01$, $^{***}p < 0.001$, $^{****}p < 0.0001$. E.V., empty vector; Uninf., uninfected.

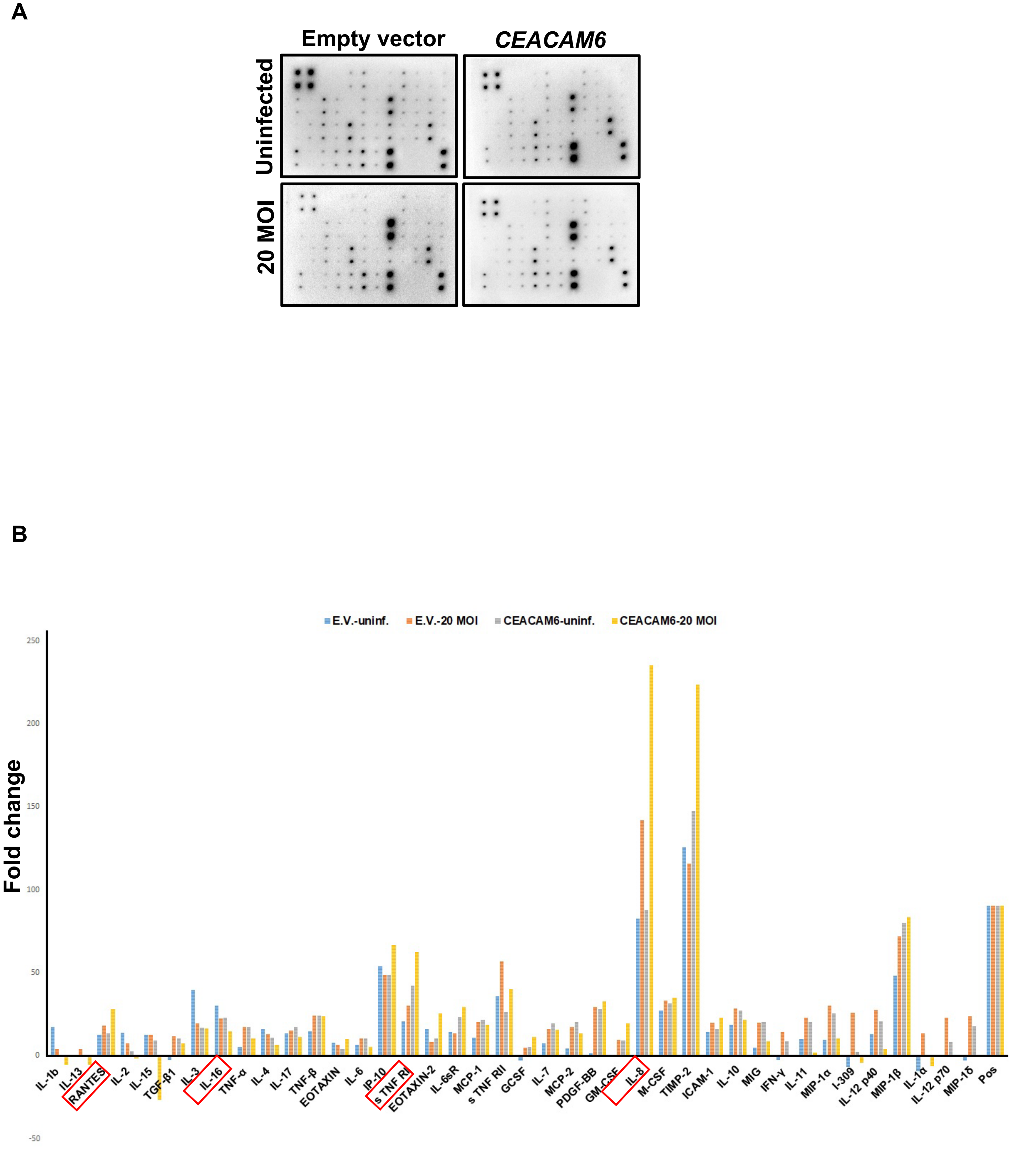

Fig. S4. Soluble mediators released by *CEACAM6*-expressing cells. (A) Immunoblots show the cytokine spots obtained from the supernatants. **(B)** The column graph shows the cytokines detected from supernatants derived from the empty vector or *CEACAM6* stably-transfected cells with or without *H. pylori* infection using the human inflammation antibody array kit from Abcam. Highlighted cytokines are associated with macrophage polarisation
